# An Elusive Case of Transient 6:1 High-Grade Atrioventricular Block Despite Normal Initial Testing

**DOI:** 10.7759/cureus.98384

**Published:** 2025-12-03

**Authors:** Colten L Witte, David J Dillard, Thomas D Mesaris, Alexis N Hall, Darren Beck, Ghassan Dalati

**Affiliations:** 1 College of Osteopathic Medicine, Edward Via College of Osteopathic Medicine, Auburn, USA; 2 Cell Biology and Physiology, Edward Via College of Osteopathic Medicine, Auburn, USA; 3 Cardiology, Medical Center Enterprise, Enterprise, USA

**Keywords:** atrioventricular block, high-grade, holter monitoring, presyncope, sleep apnea and hypertension

## Abstract

We present a case of a 55-year-old man with a prior history of syncope and vertigo due to high-grade atrioventricular (AV) block despite initial negative tests. The patient underwent multiple diagnostic evaluations, including a normal echocardiogram, myocardial perfusion imaging, Holter monitoring, and electrocardiography. As his symptoms continued to persist, an implantable loop recorder (Confirm Rx™; Abbott, Sylmar, USA) was inserted subcutaneously, which revealed transient 6:1 high-grade AV block and bradycardia. Despite normal initial investigations, his persistent symptoms and documented conduction abnormality prompted pacemaker (Abbott, Sylmar, USA) placement. This case highlights the importance of detailed electrophysiologic assessment and close monitoring in patients with recurrent unexplained syncope and high-grade AV block, even with prior normal testing.

## Introduction

High-grade atrioventricular (AV) block is a serious cardiac conduction disturbance that can cause syncope and presyncope, necessitating permanent pacemaker implantation [1]. Causes vary from structural heart disease, including idiopathic conduction system fibrosis and myocardial infarctions, to cardiotoxic compounds, such as digoxin and carbon monoxide. Essentially, any interruption in the electrical impulses of the AV conduction system can cause AV block [2]. A thorough evaluation, including resting electrocardiogram, ambulatory monitoring, stress testing, and electrophysiologic studies, is essential for diagnosis [3]. Here, we report a unique case with recurrent syncope and transient high-grade AV block, with comprehensive negative evaluations prior to pacemaker placement, highlighting the importance of ongoing assessment in symptomatic patients.

## Case presentation

A 55-year-old man presented with recurrent episodes of syncope and presyncope with a history of arthritis, hyperlipidemia, hypothyroidism, obesity (BMI 36.4 kg/m^2^), and obstructive sleep apnea (OSA) treated with a continuous positive airway pressure (CPAP) machine for years. He had no allergies and did not take any medications. His family history included a cerebrovascular accident in his brother, heart disease in his maternal grandmother, and cancer in his father, who was exposed to Agent Orange. Surgical history included a right hip replacement. 

**Figure 1:**
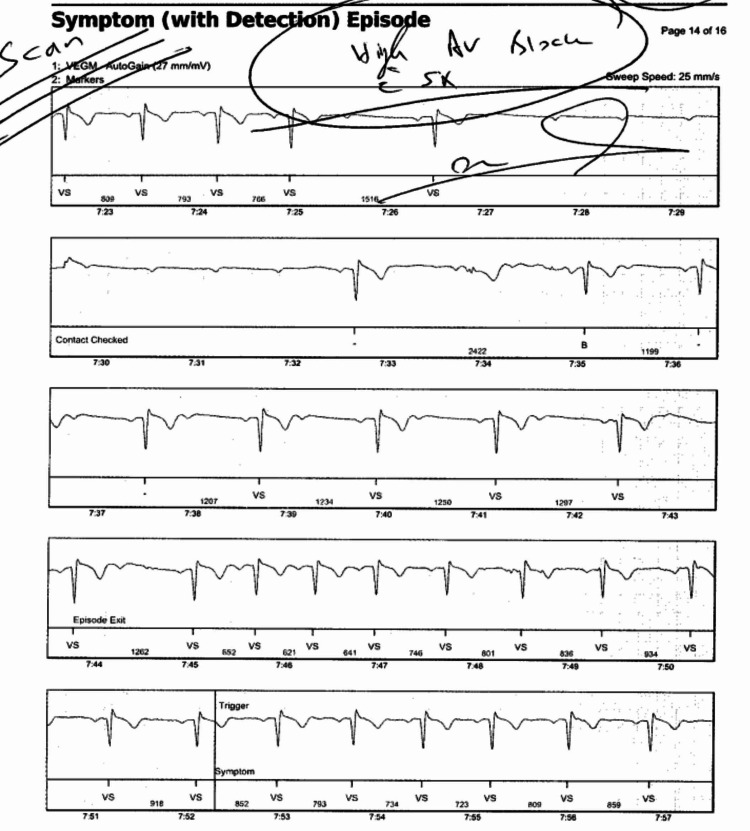
Loop recorder findings The high-grade atrioventricular block started at 7:27 (minutes:seconds) and ended at 7:32 (minutes:seconds).

Event monitoring followed by Holter monitoring was performed, and both returned negative for any conduction abnormalities, despite correct placement. Five months later, the patient returned with the same symptoms as before. An echocardiogram, implantable loop recorder (Confirm Rx™; Abbott, Sylmar, USA), and myocardial perfusion imaging test were performed, all of which were negative, despite the patient continuing to suffer episodes of dizziness and bradycardia. Another follow-up appointment was scheduled two months later, with instructions to return to the office if the symptoms worsened. Ten days later, the patient returned to the office with worsening symptoms. Upon checking the loop recorder logs, it was discovered that the patient underwent an episode of 6:1 high-grade AV block with associated presyncope the day prior, as seen in Figure 1. Due to the high-grade AV block and persistent bradycardia, pacemaker (Abbott, Sylmar, USA) placement was indicated [4]. On follow-up, the patient reported no further episodes of bradycardia, presyncope, or syncope. Regular semi-annual follow-up appointments have been scheduled.

## Discussion

High-grade AV block can be degenerative or secondary to ischemia, medication effects, or conduction system disease. It is a more severe form of second-degree AV block characterized by a loss of ventricular response to two consecutive P waves. Typical presentations of high-grade AV block follow the P wave to QRS complex and range from 2:1 to 3:1; however, in this case, the patient's ratio was high-grade and 6:1 (Figure 1), adding to the peculiarity of this case [2]. ​​Moreover, second-degree AV block is exceedingly rare at 0.18% in ostensibly healthy populations [5]. In this case, no ischemia was detected, and the absence of medication use suggests intrinsic conduction system disease. OSA is commonly associated with nocturnal bradycardia and AV conduction abnormalities, which may exacerbate underlying conduction system disease, increasing the risk of high-grade AV block [4]. 

Although the initial non-invasive tests, including Holter monitoring and stress testing, were unremarkable, the appearance of symptomatic high-grade AV block on loop recorder monitoring warranted definitive intervention with a permanent pacemaker. Current guidelines recommend pacemaker implantation in patients with symptomatic AV block higher than first-degree, regardless of initial evaluation results, especially when episodes are documented and correlate with clinical symptoms [4]. 

## Conclusions

This case highlights that normal routine assessments should not exclude rare cardiac dysrhythmias, such as transient high-grade AV block, particularly in patients with risk factors like OSA and obesity. Close follow-up with devices capable of long-term rhythm monitoring, such as loop recorders, is crucial in these scenarios. Once the diagnosis is made, prompt pacemaker placement is vital.
